# Viral Fitness of Baloxavir-Resistant Recombinant Influenza B/Victoria- and B/Yamagata-like Viruses Harboring the I38T PA Change, In Vitro, Ex Vivo and in Guinea Pigs

**DOI:** 10.3390/microorganisms11051095

**Published:** 2023-04-22

**Authors:** Amel Saim-Mamoun, Julie Carbonneau, Chantal Rhéaume, Yacine Abed, Guy Boivin

**Affiliations:** Research Center, Infectious Diseases of the CHU de Québec-CHUL, Laval University, Québec City, QC G1V 4G2, Canada

**Keywords:** influenza B, PA-I38T substitution, viral fitness, baloxavir

## Abstract

Seasonal influenza A and B viruses may cause severe infections requiring therapeutic interventions. Baloxavir, the latest antiviral drug approved against those infections, targets the endonuclease activity encoded by the polymerase acidic (PA) protein. While appearing effective at cessation of viral shedding, baloxavir demonstrated a low barrier of resistance. Herein, we aimed to assess the impact of PA-I38T substitution, a major marker of baloxavir-resistance, on the fitness of contemporary influenza B viruses. Recombinant wild-type (WT) influenza B/Phuket/2073/13 (B/Yamagata/16/88-like) and B/Washington/02/19 (B/Victoria/2/87-like) viruses and their respective PA-I38T mutants were used to evaluate replication kinetics in vitro, using A549 and Calu3 cells, and ex vivo, using nasal human airway epithelium (HAE) cells. Infectivity was also assessed in guinea pigs. In the B/Washington/02/19 background, there were no major differences between the recombinant WT virus and its I38T mutant when viral replication kinetics were evaluated in human lung cell lines and HAE as well as in nasal washes of experimentally infected guinea pigs. By contrast, the I38T mutation moderately impacted the B/Phuket/2073/13 viral fitness. In conclusion, contemporary influenza B viruses that may acquire baloxavir-resistance through the PA-I38T substitution could retain a significant level of fitness, highlighting the importance of monitoring the emergence of such variant.

## 1. Introduction

Seasonal influenza epidemics are currently caused by influenza A (H1N1 and H3N2) strains, in addition to influenza B viruses belonging to B/Yamagata/16/88 and B/Victoria/2/87 genetic lineages. Of note, strains from the B/Yamagata lineage have not circulated in the past few years [1,2]. These annual influenza epidemics declined markedly in 2020–2021 and 2021–2022 after the emergence of the SARS-CoV-2 pandemic; however, an early vigorous A(H3N2) epidemic was observed in fall of 2022 after the cessation of sanitary measures [3]. Influenza B viruses can be responsible for ≥50% influenza cases during some winter/spring epidemics, and these strains are associated with more severe diseases than influenza A viruses in children [4].

Neuraminidase (NA) inhibitors (NAI), including oseltamivir, zanamivir and peramivir, constitute a valuable complement to annual vaccines for the control of seasonal influenza infections. Clinical benefits provided by NAIs are particularly significant when the therapy is instituted within 48 h after the onset of symptoms [5]. Interestingly, influenza B strains are intrinsically less sensitive to oseltamivir, the most widely prescribed NAI, than influenza A viruses [6,7]. Immunosuppression and prolonged viral shedding are risk factors for the emergence of resistance to one or more NAIs. Indeed, the first clinical case of resistance to zanamivir occurred in an immunocompromised child infected with an influenza B virus [8].

Besides NA, influenza polymerase proteins have also been targeted for the development of anti-influenza compounds [9]. Baloxavir (BXA) is a cap-dependent endonuclease inhibitor of the influenza polymerase acid (PA) protein [10]. The active compound, baloxavir acid, blocks the initiation of mRNA synthesis through its binding with divalent cations within the active site of the endonuclease enzyme [10,11]. In cell-culture experiments, baloxavir demonstrated potent activity against seasonal influenza A and B viruses including variants harboring mutations of resistance to NAIs [10,12]. A single dose (40 or 80 mg depending on weight) of baloxavir provided a significantly greater reduction in the median duration of viral shedding than oseltamivir (24 h vs. 72 h) [13]. Unfortunately, baloxavir can also be associated with the rapid emergence of resistant variants. Clinical trials identified the I38T substitution, within the endonuclease domain of the PA protein, as the major marker of baloxavir resistance for both influenza A and B viruses [11,13,14,15].

We and others previously investigated the impact of the I38T PA substitution in A(H1N1), A(H3N2) and B strains on in vitro replicative properties and virulence/transmissibility using various animal models (mice, guinea pigs and ferrets) [11,14,16,17,18,19,20]. Depending on the viral background and the experimental design, the I38T substitution has been shown to alter or not the viral fitness of influenza A viruses, although very few reports have focused on influenza B viruses. In our previous influenza B studies, we investigated the impact of the PA-I38T substitution on the recombinant B/Phuket/2073/2013 (Yamagata-like) virus, in vitro and in experimentally infected mice [16]. More recently, we selected the PA-I38T substitution by performing cell-culture passages of an influenza B/Quebec/MCV-11/2019 (Victoria-like) isolate [20]. These experiments suggested a differential impact on fitness depending on the virus type and lineage. In the present study, we rescued contemporary B/Victoria- and B/Yamagata-like variants to determine the impact of the I38T substitution on replication kinetics, in vitro (lung cell lines), ex vivo (nasal human airway epithelium, HAE) and in guinea pigs.

## 2. Materials and Methods

### 2.1. Cell Lines and Viruses

ST6-GalI-MDCK cells overexpressing the ⍺2-6 sialic acid receptor [21] (kindly provided by Y. Kawaoka from the University of Wisconsin–Madison, WI) were maintained in minimum essential medium (MEM) with 10% fetal bovine serum (FBS) (Invitrogen, Carlsbad, CA, USA), puromycin (7.5 ug/mL) and 10% HEPES. Human pulmonary epithelial A549 cells were maintained in Dulbecco’s modified Eagle’s medium (DMEM) (Invitrogen, Carlsbad, CA, USA), supplemented with 10% FBS and 10% HEPES, and Human respiratory epithelial Calu3 cells were grown in MEM supplemented with 15% fetal bovine serum and 10% HEPES. Nasal HAE were purchased from Epithelix (Geneva, Switzerland) and cultured in an air-liquid interface with MucilAir culture medium in Costar Transwell inserts (Corning, NY, USA).

Recombinant influenza B wild-type (WT) viruses and their respective I38T PA mutants were previously generated by reverse genetics using the backbones of Victoria-like (B/Washington/02/2019, WSH19; NIBSC-19/190) [20] and Yamagata-like (B/Phuket/2073/2013; PKT13, NIBSC-14/236) [22] vaccine strains.

### 2.2. Replication Kinetics in Human Lung Cell Lines

The in vitro replication kinetics of the recombinant WT and mutant viruses were determined in 12-well plates of confluent A549 or Calu3 cells infected with a multiplicity of infection (MOI) of 0.002. Briefly, cells were washed with PBS (Phosphate buffer saline), and the infection was performed in DMEM supplemented with 0.5 μg/mL TPCK-treated trypsin (Sigma, Oakville, ON, Canada) for A549 cells and in MEM supplemented with 1 μg/mL TPCK for Calu3 cells. Supernatants were harvested at 12, 24, 48, 72, 96 and 120 h post-inoculation (p.i.) and titrated by qRT-PCR (see below) or by 50% tissue culture infective dose (TCID50) assays on ST6GalI-MDCK cells with the Reed and Muench endpoint method [23].

### 2.3. Replication Kinetics in HAE Cells

The apical poles of HAE inserts were gently washed with warm opti-MEM before infection. The epithelium was infected with an MOI of 0.002. Viruses were adsorbed at 33 °C under a 5% CO_2_ atmosphere for 30 min. Then, the inoculum was harvested, and the HAE was cultured at the air-liquid interface. Apical washes were collected at 24, 48, 96, and 120 h p.i. and titrated by quantitative RT-PCR (see below) or by TCID50 assays.

### 2.4. Viral RNA Isolation and Quantitative RT-PCR

Viral RNA extraction was performed from 90 μL of supernatants from A549 and Calu3 cells or of HAE apical washes using the MagNA Pure LC system (Total nucleic acid isolation kit, Roche Molecular System, Laval, QC, Canada). Reverse transcription quantitative PCR (qRT-PCR) assay was performed using primers targeting the influenza NS1 gene of influenza B viruses (available upon request). This assay was performed with the QuantiTect Virus + ROX Vial Kit (Qiagen, Toronto, ON, Canada) on a LightCycler^®^ 480 system (Roche Molecular System).

### 2.5. Experimental Infection of Guinea Pigs

Six- to eight-week-old female Hartley strain guinea pigs (250–300 g) (Charles River, Saint-Constant, QC, Canada) were housed in individual cages. Index animals (n = 6) were anesthetized with isoflurane and infected with an intranasal inoculum of 10^5^ PFU in a volume of 250 uL (125 uL by nostril) of the recombinant viruses. Twenty-four hours later, naïve guinea pigs (n = 3) were individually placed in the cage of an index animal to assess the potential viral transmissibility by the direct contact (DC) route. Nasal washes were collected from index and contact guinea pigs at 1, 2, 4, 6, 8 and 10 days p.i. and used to determine viral titers and viral loads by TCID50 and qRT-PCR assays, respectively.

Serum samples were collected from each guinea pig before inoculation and on day 21 p.i. to evaluate specific antibody levels against influenza B recombinants using standard HAI assays [24]. Briefly, sera were treated overnight with Receptor Destroying Enzyme (RDE II, Accurate Chemical & SC, Cedarlane) at 37 °C to remove nonspecific inhibitors. This was followed by incubation with 0.5% turkey red blood cells (Washed blood turkey alsevers, Cedarlane) at 4 °C for 60 min to remove nonspecific agglutinins. Treated sera were diluted in phosphate-buffered saline (PBS) (1:10) containing 4 hemagglutinin units of each recombinant virus. After an incubation of 30 min at room temperature, turkey red blood cells were added to the mixture, followed by another incubation of 30 to 45 min. The HAI titer was defined as the reciprocal of the last dilution that inhibited hemagglutination [24].

### 2.6. Sequencing of the Polymerase Genes

Viral PA, PB1 and PB2 genes were amplified by reverse transcription (RT)-PCR from nasal washes collected from animals on day 6 p.i. and sequenced using the ABI 3730 DNA Analyzer (Applied Biosystems, Carlsbad, CA, USA).

### 2.7. Statistical Analyses

Statistical analyses were performed with GraphPad Prism version 9.1.0. A two-way ANOVA test (with Bonferroni correction) was used to compare viral titers from replication kinetics experiments performed in A549 and Calu3 cells, HAE and nasal washes of guinea pigs.

## 3. Results

### 3.1. Impact of the PA-I38T Substitution on Replicative Capacities of Recombinant Influenza B Viruses on A549 Cells

**Figure 1:**
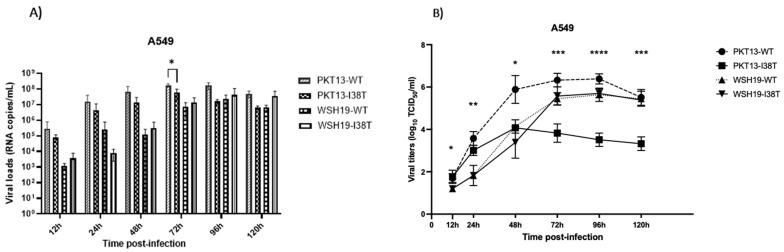
Replication kinetics of recombinant PKT13 and WSH19 viruses and their PA-I38T counterparts on A549 cells. Confluent cells were infected at an MOI of 0.002 for each virus. Supernatants were harvested at the indicated time points and used to quantify viral loads by qRT-PCR (**A**) or by TCID50 assays (**B**). Data are from two experiments, each performed in triplicate. Statistical analysis was made with a two-way ANOVA. * *p* < 0.05, ** *p* < 0.01, *** *p* < 0.001 and **** *p* < 0.0001 for differences between PKT13-WT and PKT13-I38T.

In replication kinetics experiments performed on A549 cells, PKT13-WT and PKT13-I38T recombinants reached a peak at 72 h p.i. with mean titers of 1.71 × 10^8^ and 5.98 × 10^7^ copies/mL, respectively, while WSH19-WT and WSH19-I38T reached a peak at 96 h p.i. with mean titers of 2.26 × 10^7^ and 4.17 × 10^7^ copies/mL, respectively (Figure 1A). The WSH19-WT recombinant and its I38T variant showed comparable viral loads at all time points, whereas there was a slight decrease in the mean viral RNA load for the I38T mutant in the PKT13 background compared to the WT at 72 h p.i. (5.98 × 10^7^ vs. 1.7 × 10^8^; *p* < 0.05) (Figure 1A). A sharper difference between the PKT13-WT and PK13-I38T titers was observed at all time points by TCID50 (Figure 1B). By contrast, WSH19-WT and WSH19-I38T recombinants had comparable TCID50 titers at all time points.

### 3.2. Impact of the PA-I38T Substitution on Replicative Capacities of Recombinant Influenza B Viruses on Calu3 Cells

**Figure 2:**
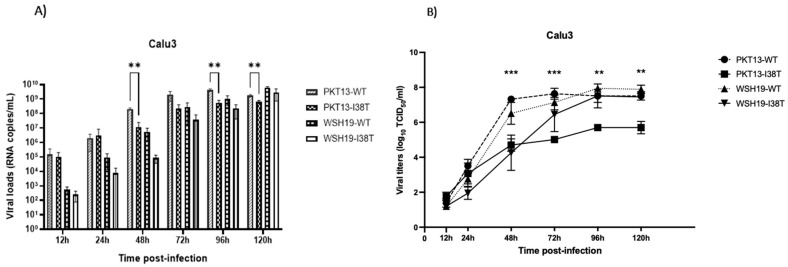
Replication kinetics of recombinant PKT13 and WSH19 viruses and their PA-I38T counterparts on Calu3 cells. Confluent cells were infected at an MOI of 0.002 for each virus. Supernatants were harvested at the indicated time points and used to quantify viral loads by qRT-PCR (**A**) or by TCID50 titrations (**B**). Data are from two experiments, each performed in triplicate. Statistical analysis was made with a two-way ANOVA. ** *p* < 0.01 and *** *p* < 0.001 for differences between PKT13-WT and PKT13-I38T.

When using Calu3 cells, PKT13-WT and PKT13-I38T recombinants reached a peak at 96 h p.i. with mean viral loads of 4.16 × 10^9^ and 5.52 × 10^8^ copies/mL, respectively, while WSH19-WT and WSH19-I38T reached a peak at 120 h p.i. with mean viral loads of 6.29 × 10^9^ and 2.88 × 10^9^ copies/mL, respectively (Figure 2A). Here, again, there was a reduced mean viral load of the recombinant PKT13-I38T versus the respective WT at 48 h p.i. (1.18 × 10^7^ vs. 2.06 × 10^8^; *p* < 0.01), 96 h p.i. (1.6 × 10^7^ vs. 1.7 × 10^8^; *p* < 0.01) and 120 h p.i. (6.33 × 10^6^ vs. 4.99 × 10^7^; *p* < 0.01) while no difference could be seen between the WSH19 recombinant viruses at any time point (Figure 2A). A similar pattern was obtained with TCID50 assays where the PKT13-I38T titers were significantly reduced compared to the PKT13-WT counterpart between 48 and 120 h p.i. with no significant difference between WSH19-I38T and WSH19-WT TCID50 titers (Figure 2B).

### 3.3. Impact of the PA-I38T Substitution on Replicative Capacities of Recombinant Influenza B Viruses in Nasal Human Airway Epithelium

**Figure 3:**
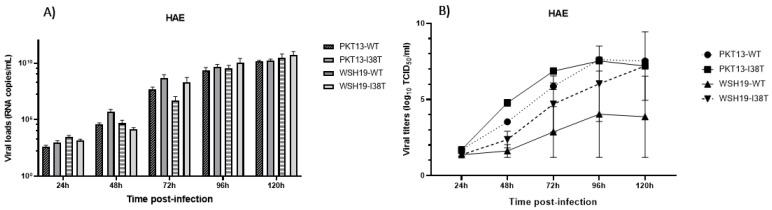
Replication kinetics of recombinant PKT13 and WSH19 viruses and their PA-I38T counterparts on nasal human airway epithelium (HAE). HAE inserts were infected at the apical pole at an MOI of 0.002 for each virus. Supernatants were harvested at the indicated time points and used to quantify viral loads by qRT-PCR (**A**) or by TCID50 assays (**B**). Data are from two experiments, each performed in triplicate. Statistical analysis was made with a two-way ANOVA.

In ex vivo replication kinetics, viruses from the two lineages generated similar viral loads at all time points in HAE (Figure 3A). There were no significant differences in viral titers between the WT and the respective I38T mutant for the two backgrounds. At the last time point (96 h), PKT13-WT and PKT13-I38T recombinants reached a peak of 1.56 × 10^10^ and 1.6 × 10^10^ copies/mL, respectively, while WSH19-WT and WSH19-I38T recombinants reached a peak of 2.96 × 10^10^ and 5.4 × 10^10^ copies/mL, respectively. Additionally, there was no significant difference in viral titers between the WT and the respective I38T variant at any time point by TCID50 assays (Figure 3B).

### 3.4. Infectivity of Recombinant Influenza B Viruses in Guinea Pigs

**Figure 4:**
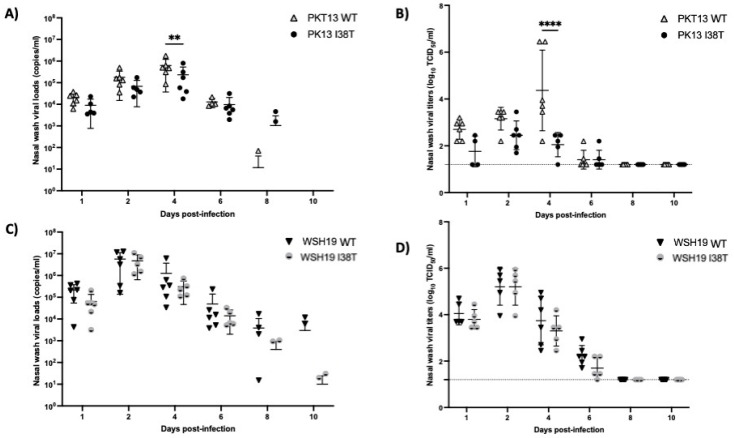
Infectivity of recombinant PKT13 and WSH19 viruses and their PA-I38T counterparts in guinea pigs. Index animals (6 per group) were inoculated with 105 PFU of recombinant viruses PKT13 or WSH19 and their PA-I38T counterparts. Nasal washes were collected at days 1, 2, 4, 6, 8 and 10 and titrated by RT-qPCR assays (**A**,**C**) and TCID50 assays (**B**,**D**). The limit of detection is shown as a horizontal dashed line for viral titers. ** *p*-value < 0.01, **** *p*-value < 0.0001.

The impact of the PA-I38T substitution was assessed in experimentally infected guinea pigs. Both infectious viral titers and viral RNA loads were determined in nasal washes from animals infected with WT and PA-I38T recombinant viruses (index) and from their direct contacts (DC) for a period of 10 days post-inoculation (p.i.). Infected guinea pigs (n = 6 per group) shed infectious viruses between days 1 and 6 p.i. for the two genetic lineages (Figure 4A–D). In the B/Yamagata lineage (PKT13), the peak viral load was reached on day 4 p.i. when the WT outgrew the PA-I38T (viral load of 6.26 × 10^5^ copies/mL compared to 2.3 × 10^5^ copies/mL (*p* < 0.01) (Figure 4A) with mean infectious viral titers on day 4 p.i. of 4.4 log_10_ TCID_50_/mL vs. 2.1 log_10_ TCID_50_/mL (*p* < 0.0001) (Figure AB), respectively). Viral titers at other time points were similar between the WT and its respective I38T mutant. At day 8 p.i, 2 of 6 guinea pigs infected with PKT13 I38T were still positive by RT-qPCR vs. only 1 animal in the PKT13 WT group.

For the B/Victoria lineage (WSH19), viral replication kinetics in guinea pigs were similar between the WT and I38T groups at all time points. The WSH-WT and PA-I38T recombinants reached their peaks at day 2 p.i. (viral loads of 5.7 × 10^6^ copies/mL and 4.8 × 10^6^ copies/mL (Figure 4C)) with mean infectious viral titers of 5.2 log_10_ TCID_50_/mL in both cases on day 2 p.i. (Figure 4D). At day 8 p.i., 2 guinea pigs infected with WSH19-I38T were still positive by RT-qPCR vs 4 animals in the WSH19-WT group. Notably, none of the DC animals (n = 3 per group) shed the virus by either cell culture or RT-PCR (not shown). Sanger sequencing of the polymerase genes from nasal wash samples collected at day 6 p.i. confirmed the presence of the I38T mutation in the PA gene without additional changes.

A seroconversion was demonstrated for all WT and PA-I38T index animals for the two lineages using HAI assays. HAI titers increased from <10 (on day 0) to 20–320 (on day 21 p.i.). Again, none of the DC animals seroconverted (HAI titers <10) (Table 1).

## 4. Discussion

Until recently, antiviral options against influenza infections were limited to NAIs. Oseltamivir is the most widely used compound from this class owing to its oral bioavailability. Because of structural differences between their NAs, influenza B viruses are less susceptible to NAIs than influenza A viruses, as reflected by higher oseltamivir IC50 values observed for old IBV isolates [25] and recently circulating IBV strains [15]. In fact, it has been suggested that oseltamivir may already have intrinsically reduced effectiveness against WT influenza B viruses, and, consequently, NA substitutions which only moderately alter the oseltamivir susceptibility of influenza B viruses may significantly impact the clinical effectiveness of the drug. Consequently, baloxavir is an important addition to the anti-influenza arsenal due to its different mechanism of action and to the convenience of its single-dose use. However, the development of resistance to this antiviral, which mainly involves the PA I38T substitution, constitutes a public health concern. Of most interest is the need to assess the fitness of the baloxavir-resistant I38T variant and its transmissibility between individuals. Research studies on this subject are particularly needed for influenza B viruses since they are generally under-represented in pre-clinical and clinical studies.

The aim of our study was to assess the impact of baloxavir resistance on contemporary influenza B viruses at different levels: in vitro, ex vivo and in vivo. The in vitro and ex vivo experiments were performed by using cells from human origin. This consists of pulmonary (A549 and Calu3) cells and nasal human airway epithelium (MucilAir) to better mimic natural lower and upper respiratory tract conditions. To assess specifically the impact of the I38T substitution, we have chosen to use recombinant viruses as the reverse genetics approach precludes eventual confounding effects of other changes elsewhere in the influenza B genome, such as mutations that may arise in cell-culture passages, in presence of BXA or during BXA treatment of patients. To our knowledge, our study is the first to investigate the infectivity of recombinant PA-I38T influenza B variants using contemporary vaccine strains of B/Victoria and B/Yamagata lineages.

In the B/Yamagata background, the PKT13-I38T variant showed reduced viral titers compared to the WT at many time points in A549 and Calu3 cells (Figure 1 and Figure 2). However, we could not make a distinction between the two recombinants in HAE experiments. This suggests that the viral fitness of influenza B viruses may vary according to the cell lines selected for evaluation. Indeed, in a previous study conducted by our group, the recombinant PKT13-WT virus and its PA-I38T variant were found to grow at comparable titers in ST6GalI-MDCK cells [16]. The same observation was made for older B/Yamagata-like (B/Maryland/1/1959) recombinants, where the WT and its I38T variant grew at similar titers at all time points in both MDCK and RPMI2650 cells [11]. In the B/Victoria context, the recombinant WSH19-I38T variant exhibited similar replication properties when compared to WSH19-WT in A549 and Calu3 replication kinetics experiments (Figure 1 and Figure 2) as well as in the ex vivo model (Figure 3).

We further assessed the impact of the I38T PA substitution on virus infectivity in vivo. We are the first group, to our knowledge, to characterize influenza B PA-I38T variants using the guinea pig model. Unlike ferrets, the gold standard animal model to study the influenza virus fitness, guinea pigs are relatively small; hence, easier to handle, inexpensive, and seronegative guinea pigs can be easily obtained, which facilitates statistical analyses [26]. Additionally, it has been shown that these animals are highly susceptible to seasonal influenza virus infection with efficient transmission of influenza A and B viruses without the need for adapting the virus priorly by serial passages [19,26]. The study design for influenza B infectivity was the same as the one that we used to evaluate the infectivity/transmissibility of the I38T PA variant in the A/H3N2 context [19]. All influenza B-infected animals seroconverted (HAI titers increased from <10 to 20–320) (Table 1) and viruses could be recovered from nasal washes collected between day 1 p.i. and day 6 p.i. (Figure 4). This experiment reinforced the impact of the I38T PA change on the fitness of the PKT13 strain (reduced viral RNA load and viral titer at day 4 p.i., Figure 4). On the other hand, our in vivo results confirm the absence of fitness cost for the BXA resistance substitution in the WSH19 background.

Unfortunately, we cannot make any conclusion regarding the impact of I38T substitution on viral transmissibility as we found no evidence for viral infection in any DC animals, based on nasal wash viral titers by culture and qRT-PCR. Accordingly, serum samples from DC animals showed HAI titers of <10, similar to pre-infection serum samples (Table 1). We hypothesize that the room temperature, here at 20 °C, in which animals were housed was inadequate for the transmission of influenza B viruses. Indeed, another group previously reported that decreasing the room temperature from 20 °C to 5 °C resulted in an important increase (from 50% to 100%) in the transmission rate in a guinea pig model of influenza B virus infection [27]. Another cause can be strain-to-strain variability of human influenza B virus transmission in guinea pigs [26]. When assessed in ferrets as pure viral populations, the I38T PA change exerted minimal reduction in contact and airborne transmission of recombinant B/Brisbane/60/2008 and B/Phuket/2073/2013 I38T viruses [28]. This was also the case for both A/H1N1 and A/H3N2 isolates [18] as well as for recombinant A/California/04/2009 (H1N1)pdm09 and A/Texas/71/2017 (H3N2) viruses [29]. A(H1N1)pdm09 and A(H3N2) I38T variants isolated from Japanese patients were found to have unaltered fitness compared to WT isolates when assessed in hamsters. In addition, these variants transmitted efficiently between ferrets through respiratory droplets [30].

Taken together, our results combined with those of others suggest that the fitness of BXA-resistant I38T B variants is unlikely to be significantly compromised in most influenza strains. Of note, the relative cost for the I38T PA change was suggested to be higher in A(H1N1)pdm09 than in A(H3N2) viruses [18].

Although the fitness of influenza B variants bearing the PA-I38T substitution seems to be mainly retained in our experiments using pure WT or I38T populations, it would also be interesting to perform competition experiments in vitro and/or in vivo by mixing both WT and PA-I38T viruses in the inoculum and analyzing the evolution of each viral population over time [18]. Studies conducted by our team showed that polymerase activity (assessed by minigenome assay) was not affected by PA-I38T change in the PKT13 background [16] as well as in a B/Victoria-like recent clinical B/Quebec/MCV-11/2019 isolate [20]. Indeed, the I38T substitution has only limited impact on the polymerase activity of most tested influenza A subtypes and B strains. However, it appeared to result in a 10-fold reduction of the endonuclease activity of influenza A and B viruses [11].

## 5. Conclusions

In conclusion, our findings confirm that the relative fitness of BXA-resistant IBVs is relatively conserved but could vary slightly according to the viral strain. It would be of interest to conduct biochemical studies with the influenza endonuclease enzyme. Our study also highlights that surveillance of BXA resistance, which could be induced at high level, is of high importance due to the relatively good fitness of the main I38T variant in the B/Victoria lineage background. Reevaluating dosing regimens and administration schedules of BXA could help reduce the rate of resistance. The combination of BXA with antivirals that have different mechanisms of action, such as a NAIs, could constitute another option to reduce the emergence of resistant variants [31]. The major limitation of our study was our inability to assess the impact of the I38T substitution on direct-contact and aerosol transmission of our contemporary influenza B recombinant viruses, which is probably due to our animal housing conditions.

## Figures and Tables

**Table 1:** Reciprocal serologic titers of index guinea pigs and their direct contacts (DC), following experimental infections with recombinant PKT13 and WSH19 wild type (WT) viruses and their PA-I38T counterparts, as assessed by hemagglutination inhibition assays.

Virus	Guinea Pig *	Day 0	Day 21
B/Phuket/2073/2013—WT	Index—1	<10	160
	Index—2	<10	320
	Index—3	<10	80
	Index—4	<10	160
	Index—5	<10	80
	Index—6	<10	80
	DC—1	<10	<10
	DC—2	<10	<10
	DC—3	<10	<10
B/Phuket/2073/2013—I38T	Index—1	<10	320
	Index—2	<10	160
	Index—3	<10	20
	Index—4	<10	160
	Index—5	<10	80
	Index—6	<10	160
	DC—1	<10	<10
	DC—2	<10	<10
	DC—3	<10	<10
B/Washington/02/2019—WT	Index—1	<10	40
	Index—2	<10	80
	Index—3	<10	40
	Index—4	<10	20
	Index—5	<10	40
	Index—6	<10	20
	DC—1	<10	<10
	DC—2	<10	<10
	DC—3	<10	<10
B/Washington/02/2019—I38T	Index—1	<10	40
	Index—2	<10	40
	Index—3	<10	80
	Index—4	<10	80
	Index—5	<10	20
	Index—6	<10	20
	DC—1	<10	<10
	DC—2	<10	<10
	DC—3	<10	<10

* Serum samples were collected from animals on day 0, before viral inoculation and on day 21 p.i.

## Data Availability

Not applicable.
